# *Clostridioides difficile* infection in the critically ill: what kind of therapy for refractory cases

**DOI:** 10.1186/s13054-020-02869-8

**Published:** 2020-04-09

**Authors:** Patrick M. Honore, Aude Mugisha, Luc Kugener, Sebastien Redant, Rachid Attou, Andrea Gallerani, David De Bels

**Affiliations:** ICU Department, Centre Hospitalier Universitaire Brugmann-Brugmann University Hospital, Place Van Gehuchtenplein, 4, 1020 Brussels, Belgium

We read with great interest the recent paper by Manthey et al. who suggest that intravenous metronidazole monotherapy may not represent an efficient treatment for *Clostridioides difficile* infection (CDI) and future studies should evaluate combination therapy [1]. Although the paper covered a range of therapeutic regimens, we would like to expand on their discussion of treatment options, especially in refractory cases which occur quite often. In a recent study of severe CDI cases, the efficacy of tigecycline was compared with standard therapy (oral vancomycin plus intravenous metronidazole) [2]. Patients treated with IV tigecycline had significantly better outcomes of clinical cure (34/45, 75.6% vs. 24/45, 53.3%; *p* 0.02), a less complicated disease course (13/45, 28.9% vs. 24/45, 53.3%; *p* 0.02), and less CDI sepsis (7/45, 15.6% vs. 18/45, 40.0%; *p* 0.009). Rates of ileus, toxic megacolon, mortality, and relapse were similar between the two groups. Given these findings, tigecycline might be considered as a potential candidate for therapeutic use in cases of CDI refractory to standard treatment [2]. Fidaxomicin is a macrocyclic antibiotic with a narrow spectrum of activity. It kills the vegetative form of CDI bacteria and binds to its spores, preventing them from germinating and producing toxin [3]. It also causes less disruption of the gut microbiota compared with vancomycin [3]. It has been recommended for cases of recurrent and refractory CDI [3]. Fecal microbiota transplantation (FMT) is a promising new therapy for the treatment of recurrent and antibiotic refractory CDI, with cure rates of over 80% in most clinical trials to date [4]. It is based on the principle that the restoration of a healthy gut microbiota will reduce the susceptibility of a patient to CDI. Nontoxigenic *C. difficile* spores protect against the colonization by toxigenic strains and halt the development of CDI; a phase II trial found very low (2%) recurrence rate within 6 weeks of treatment in patients colonized with nontoxigenic *C. difficile* spores [5]. Phase III trials are warranted to elucidate the role of this treatment option in preventing CDI [5]. We see that the clinical armamentarium is far more developed than we thought.

## Authors’ response

Carolin F. Manthey, Valentin Fuhrmann

We thank the authors for their critical review and valuable information. Indeed, we were only able to analyze treatment in patients with CDI on our ICU that was actually administered due to the retrospective nature of our study. As pointed out by Honoré et al., further phase III trials are warranted, since the aforementioned alternative therapy options have not been tested in prospective studies in critically ill patients. The aim of our study was the analysis of initial CDI therapy in ICU patients; here, we could observe that metronidazole intravenously administered was inferior to other therapies in these patients [1]. Analyzing treatment in refractory cases would be another aim and is definitely worth looking at. Tigecycline, a member of the glycylcyclines, is a broad-spectrum agent with in vitro and in vivo activity against multidrug-resistant gram-positive and gram-negative pathogens [6]. A recent meta-analysis evaluated six retrospective cohort studies, 1 prospective study, 1 case series, and 2 case reports involving tigecycline in treating severe *C. difficile* infection. An overall cure rate of 79% (95% CI 73.0–84.5%) could be observed for 186 patients in total [7]. However, studies included were heterogenous since they included observations employing tigecycline in monotherapy as well as combination therapy of tigecycline and other agents, but overall tigecycline appears as a good treatment option in severe cases of CDI. We believe that the initial CDI therapy is crucial in critically ill patients and should be administered as combination therapy, although we need prospective data to support this recommendation. Until now, most studies evaluate metronidazole and vancomycin in CDI treatment. In refractory cases, treatment is challenging and warrants further trials evaluating and comparing newer drugs such as tigecycline and fidaxomicin as well as FMT.

## Data Availability

Not applicable.

## Abbreviations

CID: *Clostridioides difficile* infection
FMT: Fecal microbiota transplantation
